# The Effects of the Pragmatic Intervention Programme in Children with Autism Spectrum Disorder and Developmental Language Disorder

**DOI:** 10.3390/brainsci12121640

**Published:** 2022-11-29

**Authors:** Tatiana Pereira, Ana Margarida Ramalho, Ana Rita S. Valente, Pedro Sá Couto, Marisa Lousada

**Affiliations:** 1 CINTESIS.UA@RISE, University of Aveiro, 3810 Aveiro, Portugal; 2 Center of Linguistics, University of Lisbon (CLUL), 1600 Lisbon, Portugal; 3 Institute of Electronics and Informatics Engineering of Aveiro, Department of Electronics, Telecommunications and Informatics, University of Aveiro, 3810 Aveiro, Portugal; 4 Center for Research and Development in Mathematics and Applications (CIDMA), Department of Mathematics, University of Aveiro, 3810 Aveiro, Portugal; 5 School of Health Sciences (ESSUA), University of Aveiro, 3810 Aveiro, Portugal

**Keywords:** neurodevelopmental disorders, autism spectrum disorder, developmental language disorder, pragmatic language intervention, preschool-age children, pragmatic intervention programme

## Abstract

Purpose: Children with Autism Spectrum Disorder (ASD) and Developmental Language Disorder (DLD) frequently face pragmatic impairments which may result in learning, socialization, and mental health difficulties, therefore early intervention is crucial. In Portugal, the Pragmatic Intervention Programme (PICP) has been recently developed and validated, but its effects are unknown. This study aims to determine the effects of the PICP on preschool-age children with ASD or DLD with pragmatic impairments. Methods: A non-randomized controlled trial has been conducted. The children (*n* = 20) were assigned to the intervention (*n* = 11) or the control group (waiting list) (*n* = 9). Each child attended 24 PICP-based intervention sessions provided by a Speech and Language Therapist in kindergarten. The primary outcome measure was a Goal Attainment Scale (GAS) rated by parents and kindergarten teachers. Secondary outcomes include parent/teacher-reported communication skills (Escala de Avaliação de Competências Comunicativas) and an assessment of the child’s general language ability (Teste de Linguagem—Avaliação da Linguagem Pré-Escolar). Results: GAS results show that all the children in the intervention group made progress. Statistically significant differences between pre- and post-intervention assessments were found for all secondary outcomes. Conclusions: The main findings suggest that the PICP improves language in preschool-age children with ASD and DLD with pragmatic difficulties. Further research is needed to analyse the effects of the PICP for each neurodevelopmental disorder individually. These results are crucial and will contribute to future research and evidence-based practice.

## 1. Introduction

Autism Spectrum Disorder (ASD) is a highly heterogeneous neurodevelopmental disorder [1]. According to the Diagnostic and Statistical Manual of Mental Disorders—Fifth Edition—Text Revision (DSM-5-TR), a diagnosis of ASD must include deficits in (1) social interaction and communication, which occurs across multiple contexts, and (2) restricted repetitive behaviors, interests, and activities [2]. The current diagnostic gold standard includes these DSM-V-TR criteria with two essential components: direct observation of child behavior by an experienced clinician—Autism Diagnostic Observation Schedule (ADOS) and an anamnestic interview with their caregivers—Autism Diagnostic Interview-Revised (ADI-R) [3]. According to Bougeard et al.’s [4] systematic review, the prevalence of ASD in Europe ranges between 0.38% and 1.55%, and current evidence supports a global increase in ASD prevalence over recent years.

Developmental Language Disorder (DLD), in turn, is a common and heterogeneous neurodevelopmental disorder that occurs during childhood [1,5] and affects 3% to 7% of children [6]. The term applies to significant difficulties in one or more language domains, in expressive and/or receptive language that affects communication and learning without an associated biomedical condition [7,8].

Pragmatic language deficits are a core feature of ASD regardless of language level or age [9,10]. Also, children with DLD may manifest difficulties in this language domain throughout childhood [11]. These difficulties have a negative impact on learning, socialization, and mental health and may persist into adulthood [12]. Therefore, early, effective, evidence-based interventions are crucial to minimizing the long-term impacts of pragmatic language impairments [13].

There are several intervention programs mentioned in the literature to improve children’s pragmatic language [14]. Some of these programs are Social Scripts [15], Social Stories [16], Comic Strip Conversations [17], Social Use of Language Program [18], Score Skills Strategy [19], Social Thinking [20], Social Communication Intervention Project (SCIP) [21], Building Blocks Program [22], JASPER [23], Mind Reading [24] and Pragmatic Intervention Program [25,26].

Over the past few decades, some research has been conducted to determine the effects of pragmatic interventions on children with neurodevelopmental disorders, especially ASD and DLD, but the inherent heterogeneity of children with pragmatic impairments impacts the evaluation of the intervention effects, which leads the researchers to develop single-subject study designs, as described in a systematic review [27]. However, an intervention cannot be considered effective based on the results of a single-subject study design, due to a lack of external validity [28]. Also, Gerber et al. [27] mentioned that the uncalculated magnitude and external validity and reliability of outcome measures were the main limitations to interpreting the improvements’ significance. Because of this, they highlight the need for further research to develop and analyze the feasibility of outcomes that report changes in children’s language use.

The need to have an outcome capable of measuring the effects of a pragmatic intervention with the potential to show clinically significant change and proximal to the intervention content, led to a recent publication by Adams and Gaile [29], in which an adapted Goal Attainment Scale (GAS) was developed and tested for children with pragmatic impairments. The authors proposed a system based on a 7-point scale to show the hypothesized steps to achievement, the expected achievement, and descriptions of achievement over and above the expected level and state that this is an acceptable and feasible primary endpoint for assessing the outcomes of a complex social communication intervention for children with pragmatic difficulties. Adams and Gaile [29] also considered that GAS may be useful for measuring outcomes of manualized interventions that require individualization and whose populations are heterogeneous.

In Portugal, the Pragmatic Intervention Programme (PICP) [25,26] is the only intervention programme developed and content-validated for preschool-age children with pragmatic impairments. It includes 11 skills, namely: (1) eye contact, (2) joint attention, (3) turn-taking, (4) communicative response, (5) communicative initiative, (6) communicative functions, (7) comprehension and expression in verbal and non-verbal communicative contexts, (8) cohesion, (9) inferential comprehension, (10) conversation, and (11) figurative language and advocates that these skills should be worked on with different communicative partners (e.g., peers, teachers) and in multiple contexts (e.g., home, kindergarten) to promote skills generalization. However, its effects are unknown and need to be established. This study aims to determine the effects of the PICP on preschool-age children with ASD and DLD with pragmatic impairments.

## 2. Methods

To determine the effects of the PICP on preschool-age children with pragmatic impairments (ASD and DLD), a non-randomized controlled trial was carried out with a non-probabilistic sample. This study was approved by the Ethics Committee of the Health Sciences Research Unit: Nursing (734/12-2020). Written informed consents were obtained from parents after receiving a detailed explanation of the study.

The participant recruitment process began by contacting several educational institutions in the Aveiro district, Portugal. After being explained the aims of the project and granting permission for its implementation, kindergarten teachers and Speech and Language Therapists (SLTs) were given clarity regarding the inclusion and exclusion criteria to identify potential participants. The inclusion criteria were: (a) a diagnosis of DLD (children were diagnosed with DLD after a comprehensive language assessment with Teste de Linguagem—Avaliação da Linguagem Pré-Escolar (TL-ALPE) [30] applied by an SLT blind to the aims of the study; pragmatic skills were evaluated with a parent/teacher reported communication skills—Escala de Avaliação de Competências Comunicativas (EACC) [31]) or ASD (clinical diagnosis provided by the neurodevelopmental pediatrician according to DSM-V criteria, ADOS, and/or ADI-R); (b) aged between 3;6 and 6;11 years; (c) European Portuguese as a native language and (d) the presence of at least two of the five criteria proving the presence of pragmatic impairments (see Supplementary Materials). Children were excluded if they were non-verbal. All identified children were subsequently observed by the first author in their natural context (educational institution) to ensure that they met the criteria for pragmatic language impairment. Eligible children were assigned to the intervention or control group. Using the G*Power version 3.1.9.4. [32,33] to determine the sample size between two groups of independent samples (effect size: 1.76—based on the only Portuguese intervention study with children with DLD [34]; *α* = 0.05; power = 0.95; allocation ratio = 1), the result obtained was 20 (10 per group).

Accordingly, a total of 20 children were considered eligible and therefore were allocated to the intervention (*n* = 11) or control (waiting list) group (*n* = 9). The children were evaluated at two different time points: pre-intervention assessments (T1) were completed after the group allocation and post-intervention assessments (T2) were carried out within one week after the completion of the intervention/control period. Both assessments occurred in the children’s educational institution.

### 2.1. Intervention

The PICP is a manualized intervention programme that aims to promote pragmatic language skills among preschool-age children with pragmatic impairments. This intervention manual provides goals, procedures, and a description of the intervention activities to carry out in order to improve several pragmatic language skills [26] that were mentioned in the last paragraph of the introduction section. Previous research reports an achieved content validity index of 1 [25,26].

The intervention content was derived from the PICP but customized individually for each child. All children received the same number of sessions (24), previously determined after a literature review. The 24 PICP-based intervention sessions were freely given biweekly, for one hour, by one SLT (first author) with in-depth knowledge about the programme content and implementation, and previous clinical practice providing intervention to children with pragmatic impairments in educational settings. All sessions were provided face-to-face in a naturalistic context (kindergarten) and, beyond the child and the SLT, other communicative partners were also involved (e.g., peers, kindergarten teachers) in carrying out the activities to promote skills generalization, relationships, and inclusion. Before the intervention, collaborative goal setting between parents and kindergarten teachers was considered essential to tailor and prioritize intervention goals according to each child’s needs. The adopted procedure can be seen in Supplementary Materials.

The children that were allocated to the control group were on a waiting list and did not receive intervention until the T2 assessment. However, after the post-intervention assessment of the intervention group, the same intervention procedures previously defined for this group were applied and the control group also received treatment.

### 2.2. Outcome Measures

The primary outcome measure was the GAS. Before the intervention (T1), the child’s needs and the parents’ and kindergarten teachers’ priorities were mapped on to the skills addressed in the PICP and then three appropriate goals were jointly selected (see Supplementary Material Figure S1). The pragmatic language skills that were selected for each child and, therefore, on which the GAS focused, can be seen in Supplementary Material Table S2. A set of criteria to guide the writing of improvement levels for each goal, proposed by Adams and Gaile [29], was followed. An example is provided in Supplementary Material Table S3. The parents and kindergarten teachers rated the achievement of the goals at the end of the intervention period. After intervention (T2), parents and kindergarten teachers rated each goal on a 7-point scale (−1 to +5) compared to T1 (baseline) according to the child’s progress. A score of −1 indicated that the skills were below baseline; a score of 0 indicated no change from baseline; improvement to +1 was related to the child’s understanding of the target skill or the impact of any use of the skill; improvement to +2 was recorded when the child used the new skill with scaffolding and substantial support; improvement to +3 indicated achievement of the goal as expected with no or minimal support; improvement to +4 indicated generalization of the target skill to contexts other than those embedded in the intervention; and improvement to +5 indicated generalization of the target skill to additional contexts and with increasing frequency or complexity or a sense of stable and reliable use of the new skill. Improvement to the expected level would be represented by a score of +3 across all three goals with a total score of 9. A total score of 10 or higher, therefore, indicates achievement above the expected level [29].

Secondary outcomes were: (1) EACC [31]: an assessment scale used to evaluate the family’s and teachers’ perceptions of the child’s pragmatic skills, specifically in the following areas: (I) communicative intentions; (II) conversational skills; (III) responsiveness in communicative contexts; (IV) comprehension in communicative contexts; (V) coherence; (VI) cohesion; (VII) non-literal language comprehension; and (VIII) extralinguistic aspects. The scale consists of 45 items. Items are rated on a Likert-type scale with the following descriptors: never; rarely; sometimes; and often. Quantitative rating is done as follows: assigned 1 point for never, 2 for rarely, 3 for sometimes, and 4 for often. This scale should be completed by an adult who regularly accompanies the child (e.g., mother/father, kindergarten teacher) and was validated for the Portuguese population. Each parent/teacher filled out the scale individually, without knowledge of each other’s answers; (2) TL-ALPE [30]: a (Portuguese) standardized instrument that assesses semantic and morphosyntactic language skills in preschool-age children. This evaluation was undertaken by an SLT, blind to the aims of the study. Both assessments were carried out at T1 and T2 (along with determining eligibility, assessment results were also used to determine baseline and each child’s needs, as well as to monitor intervention progress).

### 2.3. Statistical Analysis

Once all the evaluations were completed, the data were entered into the Statistical Package for Social Sciences software (IBM SPSS Statistics, v28.0, Armonk, NY, USA: IBM Corp.), and analyzed considering descriptive and inferential statistics. Regarding descriptive statistics, means (M), standard deviations (SD), and 95% Confidence Interval (95%CI) were calculated for quantitative variables and percentages (%) for qualitative variables. For inferential statistics, given the small sample size, the non-parametric test Mann-Whitney U was used for group comparisons. For correlation analysis, Spearman’s rank correlation coefficient (r) was used. The Effect Size (ES) was determined by Hedge’s G. The results were considered significant if *p* < 0.05.

## 3. Results

The participants came from the same demographical region and shared similar socioeconomic backgrounds. Table 1 shows sociodemographic characteristics at T1.

The primary outcome measure was GAS. Table 2 shows parents’ and kindergarten teachers’ ratings for GAS for each participant in the intervention group. The number of goals that met expectations is also shown. The mean total score rated by parents was 11.4 ± 3.1. For teachers’ ratings, the mean total score was 12.4 ± 2.5. In both ratings, it is observed that at least two of the three goals met expectations. 

Secondary outcome measures at T2 are presented in Table 3. It is important to mention that, at T1, no statistical differences were found between the groups. The non-parametric Mann-Whitney U test was used for group comparisons between the intervention and control groups (considering the column that corresponds to the difference between the two considered moments (Difference (T2-T1)) of Table 3. The results obtained were statistically significant for EACC-P Difference (U = 4.50, *p* < 0.001), EACC-T Difference (U = 4.00, *p* < 0.001), and TL-ALPE Difference (U = 19.50, *p* = 0.021). Accordingly, a large to very large effect was observed on TL-ALPE Difference (ES = 1.070) and EACC-P Difference (ES = 1.188), and a huge effect was found for EACC-T Difference (ES = 2.293). The rules of thumb proposed by Cohen [35] and Sawilowsky [36] were used to define the effect. Additionally, a strong and statistically significant correlation was observed between EACC-P Difference and EACC-T Difference (*r* = 0.731, *p* < 0.001) and between EACC-T Difference and TL-ALPE Difference (*r* = 0.835, *p* < 0.001). For EACC-P and TL-ALPE, the correlation was moderate and statistically significant (*r* = 0.577, *p* = 0.008).

## 4. Discussion

This study aimed to establish the effects of the PICP on preschool-age children with ASD and DLD, compared to no treatment, and analyze whether this intervention also improved general language ability. This study presents important findings that might influence clinical practice and research in this field. 

Considering Adams’ and Gaile’s [29] proposal, the GAS was used as the primary outcome measure. The GAS involved the joint selection of three goals for each child before the intervention. Each goal achievement was rated after the intervention with a 7-point scale, by parents and kindergarten teachers. Considering that all children started from baseline (0), if a goal was rated +3 or higher (indicated achievement of the goal as expected with no or minimal support) it was considered to have met expectations [29]. After 24 PICP-based intervention sessions, the GAS results show that all children in the intervention group (100%) made progress and met expectations for at least two of three goals. Parents and kindergarten teachers identified that three goals have met expectations in 72.7% of cases. In Adams’ and Gaile’s [29] study, a GAS with a 7-point scale was also used and parents and professionals rated the individualized goals. Parents identified that three goals (maximum) met expectations in only 10% of cases. For professionals, three goals met expectations in 30% of cases. Although its purpose was not to determine the effects of an intervention, the results can be compared with our study, since an intervention focusing on pragmatics was applied and the GAS was only rated after the intervention. We believe that the differences between the two studies may be related to the nature of the pragmatic language skills worked on and the correspondent age group, so that skills intervened in preschool-age children may be acquired more quickly than other, more complex ones, worked on in school years.

The EACC was used as a secondary outcome measure. This measure shows a statistically significant difference between T2 and T1 in both parents’ and teachers’ ratings (intervention group) when compared to the control group. The high standard deviation that is observed in the EACC-P Difference is due to one child who had a greater improvement than the others from the parents’ perspective (this child improved from a score of 74 (T1) to a score of 153 (T2)). This variability within the intervention group may be related to the small sample size but also the heterogeneity of the ASD and DLD [1]. Additionally, it is important to mention the large to very large effect on EACC-P difference and the huge effect on EACC-T difference that was observed, which indicates that this intervention has a real impact on children’s lives. In a similar study, Adams et al. [21] used a standardized parent-rated communication checklist—Children’s Communication Checklist—2 (CCC-2) [37]—specifically CCC-PRAG (18-item pragmatic rating scale related to intervention, derived from CCC-2), and no significant intervention effects were found immediately after the intervention. However, significant changes were verified at the follow-up.

A secondary outcome measure to assess general language ability (TL-ALPE) was also used to analyze the possible influence of the intervention on other language domains. A statistically significant difference was found which indicates that the children in the intervention group have generalized their achievements and that differences in general language ability were observable. The use of standardized outcome measures with different contents than those provided in the intervention has been reported in the literature. Accordingly, in Adams et al.’s [21] study, the Clinical Evaluation of Language Fundamentals—4 (which also measures general language ability) was used as the primary outcome measure, following intervention with children with pragmatic language difficulties, and no statistical significant differences were verified.

The overall findings suggest that the PICP improves language in preschool-age children with ASD and DLD with pragmatic impairments, but further research is needed to establish the effects of this programme for each neurodevelopmental disorder individually. The GAS is a measure completely adapted to each child’s and family’s individual needs and priorities, allowing for a more specific, real analysis of a heterogeneous population. However, statistically significant results were also found in parent/teacher-reported outcomes and in a standardized instrument that assesses general language ability, which is indicative of generalization. Furthermore, beyond the content of the intervention itself, we believe that the context in which the intervention was given as well as the inclusion of multiple communicative partners may have contributed to the positive achieved outcomes and therefore must be valued.

One of the study’s limitations is the small sample size. Due to the COVID-19 pandemic, it was difficult to provide the intervention to a larger number of children since this intervention is carried out in a natural context (kindergarten). On the other hand, DLD and ASD are highly heterogeneous neurodevelopmental disorders, which can make it difficult to generalize the results. In the future, it is our intention that this study will continue to increase the sample size and allow the analysis of the results for each neurodevelopmental disorder individually to understand if there are differences between the effects of the PICP on DLD and ASD. Furthermore, our findings highlight the importance of using the GAS as an outcome measure when working with children with pragmatic impairments and value it as a key measure to consider when evaluating intervention effectiveness.

## Figures and Tables

**Table 1:** Sociodemographic characterization.

Children	Intervention Group (*n* = 11)			Children	Control Group (*n* = 9)		
	Age (months)	Condition	Gender		Age (months)	Condition	Gender
1	42	DLD	Male	12	48	DLD	Female
2	62	DLD	Female	13	65	DLD	Male
3	53	DLD	Male	14	53	DLD	Male
4	53	ASD	Female	15	56	ASD	Male
5	47	ASD	Male	16	48	DLD	Female
6	51	ASD	Male	17	67	DLD	Male
7	59	ASD	Female	18	58	DLD	Female
8	57	DLD	Male	19	62	ASD	Female
9	53	ASD	Female	20	42	ASD	Male
10	48	ASD	Female				
11	53	ASD	Male				
Total	52.5 ± 5.6	DLD (36.4%)	Male (54.5%)		55.4 ± 8.5	DLD (77.8%)	Male (55.6%)

Legend: ASD—Autism Spectrum Disorder; DLD—Developmental Language Disorder.

**Table 2:** Post-intervention (T2) results of the Goal Attainment Scale, rated by parents and kindergarten teachers.

Children *	Parents Rating					Kindergarten Teachers Rating				
	Goal 1	Goal 2	Goal 3	Total **	Goals that met expectations ***	Goal 1	Goal 2	Goal 3	Total **	Goals that met expectations ***
1	4	3	4	11	3 (100%)	3	4	4	11	3 (100%)
2	5	5	5	15	3 (100%)	5	5	5	15	3 (100%)
3	3	3	1	7	2 (66.6%)	4	4	1	9	2 (66.6%)
4	5	5	5	15	3 (100%)	5	5	5	15	3 (100%)
5	3	4	4	11	3 (100%)	3	4	5	12	3 (100%)
6	4	3	1	8	2 (66.6%)	4	4	1	9	2 (66.6%)
7	4	4	5	13	3 (100%)	4	4	5	13	3 (100%)
8	5	5	5	15	3 (100%)	3	2	4	9	2 (66.6%)
9	3	4	3	10	3 (100%)	5	5	5	15	3 (100%)
10	4	4	5	14	3 (100%)	5	5	5	15	3 (100%)
11	1	3	3	7	2 (66.6%)	5	3	5	13	3 (100%)
Mean scores	3.7 ± 1.2	3.9 ± 0.8	3.7 ± 1.6	11.4 ± 3.1	2.7 ± 0.5	4.2 ± 0.9	4.1 ± 0.9	4.1 ± 1.6	12.4 ± 2.5	2.7 ± 0.5

Legend: * intervention group; ** maximum = 15; *** maximum = 3.

**Table 3:** Pre-intervention (T1) and post-intervention (T2) results of secondary outcomes (EACC and TL-ALPE).

Outcomes	T1		T2		Difference (T2-T1)		
	Intervention (*n* = 11)	Control (*n* = 9)	Intervention (*n* = 11)	Control (*n* = 9)	Intervention (*n* = 11)	Control (*n* = 9)	95%CI
EACC-P	85.4 ± 22.8	97.9 ± 23.7	106.6 ± 31.3	98.9 ± 23.7	21.2 ± 21.3 *	1.0 ± 5.3 *	4.8–35.5
EACC-T	75.6 ± 16.2	85.1 ± 16.3	98.9 ± 20.3	85.8 ± 19.0	23.3 ± 11.1 *	0.7 ± 6.8 *	13.7–31.5
TL-ALPE	35.73 ± 27.2	45.4 ± 21.1	52.2 ± 31.1	50.3 ± 23.6	16.5 ± 13.2 *	4.9 ± 4.7 *	1.8–21.3

Legend: * *p* < 0.05; T1—pre-intervention evaluation; T2—post-intervention evaluation; CI—Confidence Interval; EACC-P—Escala de Avaliação de Competências Comunicativas rated by parents; EACC-T—Escala de Avaliação de Competências Comunicativas rated by teachers; TL-ALPE—Teste de Linguagem—Avaliação da Linguagem Pré-Escolar.

## Data Availability

The datasets generated during and/or analyzed during the current study are available from the corresponding author upon reasonable request.

## Supplementary Materials

The following supporting information can be downloaded at: https://www.mdpi.com/article/10.3390/brainsci12121640/s1, Figure S1: Example of how parents’ and teachers’ priorities lead to jointly selected goals whose achievement was rated by them; Table S1: Criteria that prove the presence of pragmatic impairments adapted from [21]; Table S2: Selected competencies per goal; Table S3: Example of a Goal Attainment Scale from the Pragmatic Intervention Programme adapted to a child’s individual needs.
