# Supplementary material for: The Effects of the Pragmatic Intervention Programme in Children with Autism Spectrum Disorder and Developmental Language Disorder
Source: Brain Sci. 2022 Nov 29;12(12):1640. doi: 10.3390/brainsci12121640 (PMC9775383; doi:10.3390/brainsci12121640)
Supplement: Supplementary file 1 [file brainsci-12-01640-s001.zip › brainsci-2027291-supplementary.pdf]

Supplementary Materials

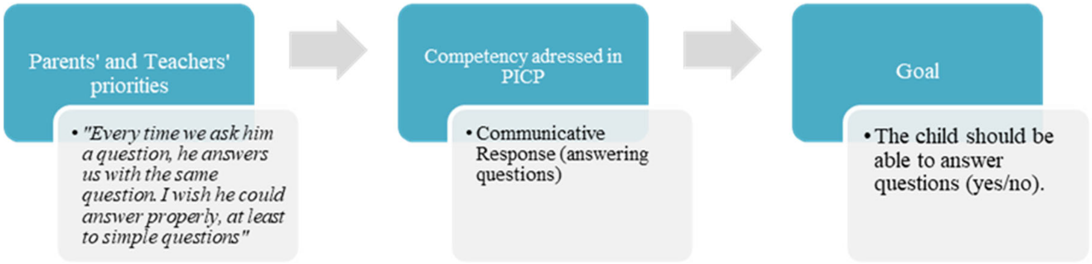

Figure S1. Example of how parents' and teachers' priorities lead to jointly selected goals whose achievement was rated by them.

Table S1. Criteria that prove the presence of pragmatic impairments adapted from [21].

i. Difficulties interacting and using language for social purposes (e.g., greeting people appropriately, making requests, requesting information, giving information, answering questions, getting attention, or starting a conversation) with peers and/or adults.	☐
ii. Difficulties understanding and/or expressing non-verbal communicative aspects (e.g., understanding facial expression; using tone of voice) and using them as cues to regulate interaction.	☐
iii. Conversational difficulties (i.e., in initiating, maintaining, or changing the topic of conversation appropriately).	☐
iv. Difficulties adapting language to the context, communicative partners, and multiple communicative situations (e.g., in making appropriate remarks).	☐
v. Difficulties making linguistic inferences or understanding non-literal language (e.g., understanding indirect requests; ironic expressions).	☐

The child must present at least two of five predefined criteria (adapted from [21].

Table S2. Selected competencies per goal.

Participants	Goal 1	Goal 2	Goal 3*
1	Communicative Response	Communicative Initiative	Communicative Functions
2	Joint Attention	Communicative Response	Communicative Functions

**3**	Communicative Response	Communicative Initiative	Communicative Functions
**4**	Joint Attention	Communicative Response	Communicative Functions
**5**	Joint Attention	Turn-Taking	Communicative Functions
**6**	Communicative Response	Communicative Initiative	Communicative Functions
**7**	Joint Attention	Communicative Response	Communicative Functions
**8**	Joint Attention	Communicative Response	Communicative Functions
**9**	Communicative Response	Communicative Functions	Negotiation
**10**	Turn Taking	Communicative Response	Communicative Functions
**11**	Communicative Response	Communicative Initiative	Communicative Functions

* The following communicative functions were instrumental and informative.

Table S3. Example of a Goal Attainment Scale from the Pragmatic Intervention Programme adapted to a child's individual needs.

Please read and mark with a cross (X) the statement that, in your opinion, best describes the child's progress toward the above goal.

Goal 1: The child should be able to answer questions (yes/no).

**+5 =** The child improved his ability to answer questions (yes/no) in real communicative situations, in more than one additional context, and with more than one communicative partner.	☐
**+4 =** The child improved his ability to answer questions (yes/no) in real communicative situations, in an additional context, and with a single communicative partner.	☐
**+3 =** The child improved his ability to answer questions (yes/no) in real communicative situations, in a specific context, and with a single communicative partner.	☐

**+2 =** The child improved his ability to answer questions (yes/no) in real communicative situations with the aid of visual stimuli.	☐
**+1 =** The child improved his ability to answer questions (yes/no) in triggered situations.	☐
**0 =** There were no changes in the response to questions (yes/no).	☐
**-1 =** The child's ability to respond to questions (yes/no) has worsened.	☐

Goal 2: The child should be able to make a request, through gestures or verbalizations.

**+5 =** The child improved his ability to make a request in real communicative situations, in more than one additional context, and with more than one communicative partner.	☐
**+4 =** The child improved his ability to make a request in real communicative situations, in an additional context, and with a single communicative partner.	☐
**+3 =** The child improved his ability to make a request in real communicative situations, in a specific context, and with a single communicative partner.	☐
**+2 =** The child improved his ability to make a request in real communicative situations with the aid of verbal or visual stimuli.	☐
**+1 =** The child improved his ability to make a request in triggered situations.	☐
**0 =** There were no changes in the ability to make a request.	☐
**-1 =** The child's ability to make a request has worsened.	☐

Goal 3: The child should be able to verbally negotiate.

**+5 =** The child has improved his ability to verbally negotiate in more than one additional context, and with more than one communicative partner.	☐
**+4 =** The child improved his ability to verbally negotiate in real communicative situations, in an additional context, and with a single communicative partner.	☐

**+3 =** The child improved his ability to verbally negotiate in real communicative situations, in a specific context, and with a single communicative partner.	☐
**+2 =** The child improved his ability to verbally negotiate in real communicative situations with the aid of verbal or visual stimuli.	☐
**+1 =** The child improved his ability to verbally negotiate in triggered situations.	☐
**0 =** There were no changes in the ability to negotiate verbally.	☐
**-1 =** The child's ability to negotiate has worsened.	☐

References

21. Adams, C.; Lockton, E.; Freed, J.; Gaile, J.; Earl, G.; McBean, K.; Nash, M.; Green, J.; Vail, A.; Law, J. The Social Communication Intervention Project: A randomized controlled trial of the effectiveness of speech and language therapy for school-age children who have pragmatic and social communication problems with or without autism spectrum disorder. *Int. J. Lang. Commun. Disord.* **2012**, *47*, 233–244.
